# Host genetic variation and HIV disease: from mapping to mechanism

**DOI:** 10.1007/s00251-017-1000-z

**Published:** 2017-07-10

**Authors:** Vivek Naranbhai, Mary Carrington

**Affiliations:** 1 Cancer and Inflammation Program, Leidos Biomedical Research Inc., Frederick National Laboratory for Cancer Research, Frederick, MD USA; 2 Ragon Institute of MGH, MIT and Harvard, Boston, MA USA; 3 Centre for the AIDS Programme of Research in South Africa (CAPRISA), University of KwaZulu-Natal, Durban, South Africa

**Keywords:** HIV, Host genetics, HLA, CCR5, Immunology

## Abstract

This review aims to provide a summary of current knowledge of host genetic effects on human immunodeficiency virus (HIV) disease. Mapping of simple single nucleotide polymorphisms (SNP) has been largely successful in HIV, but more complex genetic associations involving haplotypic or epigenetic variation, for example, remain elusive. Mechanistic insights explaining SNP associations are incomplete, but continue to be forthcoming. The number of robust immunogenetic correlates of HIV is modest and their discovery mostly predates the genome-wide era. Nevertheless, genome-wide evaluations have nicely validated the impact of HLA and CCR5 variants on HIV disease, and importantly, made clear the many false positive associations that were previously suggested by studies using the candidate gene approach. We describe how multiple HIV outcome measures such as acquisition, viral control, and immune decline have been studied in adults and in children, but that collectively these identify only the two replicable loci responsible for modifying HIV disease, CCR5, and HLA. Recent heritability estimates in this disease corroborate the modest impact of genetic determinants and their oligogenic nature. While the mechanism of protection afforded by genetic variants that diminish CCR5 expression is clear, new aspects of HLA class I-mediated protection continue to be uncovered. We describe how these genetic findings have enhanced insights into immunobiology, been clinically translated into CCR5 antagonists, allowed prioritization of antigens for vaccination efforts, and identified targets for genome-editing interventions. Finally, we describe how studies of genetically complex parts of the genome using new tools may begin revealing additional correlates.

**Electronic supplementary material:**

The online version of this article (doi:10.1007/s00251-017-1000-z) contains supplementary material, which is available to authorized users.

## Introduction

Nearly 78 million people have been infected by HIV, primarily through sexual acquisition (about 80%), and 35 million have died from HIV-1 and related diseases. HIV infects infants through mother-to-child-transmission (MTCT) and adults through bloodborne or sexual transmission. The HIV epidemic has affected subpopulations in heterogeneous fashions, with rates varying by age, sex, predominant mode of exposure (intravenous or sexual), and geographic location. The overall risk of transmission of HIV is thought to be low, roughly 0.18% per sex act, but varies considerably from 0.02 to 1.6% in published meta-analyses (Boily et al. 2009). Similarly, level of viremia during acute HIV infection, setpoint viral load, rate of CD4+ T cell depletion, and eventual rates of progression to AIDS all vary widely. For example, while some individuals have been observed to develop profound CD4+ T cell lymphopenia and AIDS within 2 years, others remain AIDS-free for up to 15 years following HIV seroconversion (van der Helm et al. 2014; Mlisana et al. 2014).

Since the sequencing of the human genome in 2001 (Lander et al. 2001), large-scale genetic and genomic studies have played an increasingly important role in delineating the pathogenesis of human disease. Understanding the variable risk and course of HIV infection in vivo by studying host genetics has been instrumental to understanding the immunopathogenesis of HIV. Moreover, it has complemented the in vitro understanding of the biology of HIV infection and replication that led to development of antiretroviral drugs, which are the cornerstone of HIV prevention and therapy.

Studies of host genetic correlates have reflected the wide-ranging nature of possible HIV outcome measures over the course of HIV infection, beginning with acquisition through disease progression and development of opportunistic infection and AIDS. Similarly, these studies reflect the heterogeneity in populations affected by HIV. Hundreds of studies have been conducted into the genetic determinants of HIV acquisition and disease progression. Older studies, referred to as candidate gene studies, were locus or gene-centric, while larger recent studies have searched for correlates across the genome using genome-wide genotyping or whole exome/genome sequencing in an unbiased, “agnostic” manner. In total, five genome-wide association studies (GWAS) of HIV acquisition (Johnson et al. 2015; Lingappa et al. 2011; Petrovski et al. 2011; Joubert et al. 2010; McLaren et al. 2013) and 11 of markers of HIV disease course have been reported (Lingappa et al. 2011; Fellay et al. 2009; Fellay et al. 2007; Herbeck et al. 2010; H. I. V. C. S. International 2010; Le Clerc et al. 2009; Limou et al. 2009; Pelak et al. 2011; Pelak et al. 2010; Troyer et al. 2011; Wei et al. 2015). Combining most of these studies, a meta-analysis in individuals of European ancestry of HIV acquisition (McLaren et al. 2013) and HIV viremia (McLaren et al. 2015) has also been reported. A range of candidate gene and GWA studies of HIV-related traits have been reported, such as atherosclerosis (Shrestha et al. 2010), response to antiretroviral drugs (Chantarangsu et al. 2011; Irvin et al. 2011; Leger et al. 2014; Lehmann et al. 2015), and blood counts in HIV-infected or -exposed patients (Ferreira et al. 2010; Moore et al. 2015; Ramsuran et al. 2011). Similarly HIV-associated diseases have also been interrogated, such as HIV-dementia (Levine et al. 2012) and hepatitis C (Ulveling et al. 2016). Although we do not discuss each study in detail here, Supplementary Table 1 provides a recent extract from the GWAS catalog for all HIV and related traits. These modern studies, in which confounding due to population structure can be adequately adjusted for, have been illuminating in refuting candidate gene findings likely to have been false-positive associations and in confirming two loci that were identified in candidate gene studies as important in modifying HIV outcomes: those encoding CCR5, a co-receptor for HIV entry, and human leukocyte antigen (HLA) class I, the protein system responsible for antigen presentation to immune cells. These GWAS have also been instrumental in generating estimates of the relative contribution of host and viral genetic variation in HIV. Accordingly, we review this literature briefly, and then turn attention to the two well-validated genetic correlates of HIV acquisition and HIV disease outcome. We specifically highlight questions, which, in our view, remain unanswered with regard to the mechanism of HLA effects in HIV. We anticipate further progress in these areas and discuss additional future prospects in this review.

### The relative role of host and viral genetic variation in HIV

Classically, twin and family studies have been used to infer the host genetic contribution to disease risk. Such studies have been exceedingly challenging to perform in HIV either with regard to HIV acquisition or HIV disease progression due to poor sample sizes, although one very enlightening sibling study has been reported, emphasizing the importance of the major histocompatibility complex (MHC) in disease outcome (Kroner et al. 1995). A second important determinant of disease course, unique to diseases caused by a polymorphic pathogen, is the relative importance of pathogen variation. While estimates of the host genetic contribution to HIV acquisition have not been reported (but likely to be modest), estimates of the genetic contribution to HIV control after infection suggest that around one third of the variance in setpoint viremia can be attributed to viral variation (Fraser et al. 2014). Modern approaches that estimate the host polygenic heritability using genome-wide genotyping data have recently been reported in HIV. In a meta-analysis of 6315 individuals for whom setpoint viremia was recorded, ~25% of variance in setpoint viremia was attributable to common host genetic variation, and the majority of this could be explained by variation in the HLA or CCR5 loci leaving around 5.5% of variance attributable to variation outside of these two regions (McLaren et al. 2015). Moreover, Bartha et al. (2013, 2017) demonstrated in studies, where viral and host genetic variation were both available, that almost all of the effect of the HLA region on setpoint viremia is mediated through its pressure on viral genetic variation, and that human variants only in the HLA region and not elsewhere in the genome are associated with specific viral mutations. These findings corroborate earlier findings of HLA-mediated selection of viral variants in vivo (Brumme et al. 2007; Carlson and Brumme 2008). Taken together, these studies are striking in demonstrating that the CCR5 and HLA loci explain the majority of host genetic effects on HIV viral control. Larger studies may yet yield additional rare variants that alter disease, but more extensive coverage through whole-genome studies has not as yet yielded new insights (Pelak et al. 2011). Variants with population-specific effects may be identified, as is suggested by early GWAS in Asian populations, for example (Wei et al. 2015). Strikingly, the genetic mapping of HIV is amongst the most complete of any complex disease, as there are few other diseases where the majority of variance in genetically mediated modification of disease course has been mapped and attributed to specific genes. Efforts in HIV genetic research have therefore focused on delineating the nature of CCR5 or HLA-mediated protection and finding therapeutic opportunities to exploit this.

### Variants in CCR5 that diminish expression of a co-receptor used for cell entry by HIV protect from acquisition and reduce rates of disease progression

**Fig. 1:**
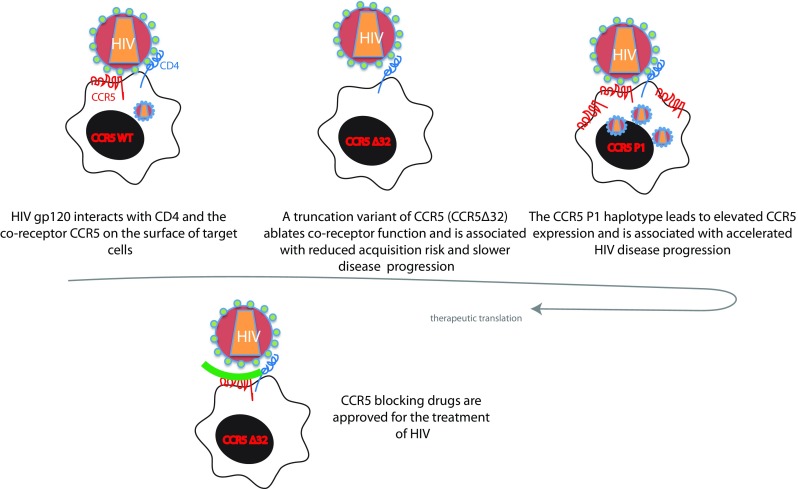
CCR5 is a key co-receptor for HIV entry. CCR5Δ32 individuals in whom CCR5 expression is lost have reduced HIV acquisition risk and disease progression while individuals with the CCR5P1 haplotype have higher CCR5 expression and accelerated disease progression. These and other observations led to drugs that block CCR5, which are licensed for the treatment of HIV

Genetic variants in CCR5, a chemokine receptor physiologically responsible for binding RANTES, MIP-1α, and MIP-1β, affect HIV acquisition risk and disease progression rates (Fig. 1). The observation that homozygosity for a 32-base pair deletion in the coding region of CCR5 (CCR5Δ32) accounted for reduced HIV infection risk was independently reported by three groups (Dean et al. 1996; Liu et al. 1996; Samson et al. 1996) within months of the identification of CCR5 as an important co-receptor for HIV viral entry (Choe et al. 1996; Deng et al. 1996; Doranz et al. 1996; Dragic et al. 1996; Rucker et al. 1996; Alkhatib et al. 1997). The frequency of this variant is the highest amongst people of European descent in whom ~10% of individuals carry a copy of the CCR5Δ32 allele, intermediate amongst those of Middle Eastern and Asian descent (2–5%), and near absent amongst individuals of African ancestry (Martinson et al. 1997). Although initial studies noting a gradient in allele frequency in Europe (Lucotte and Mercier 1998) led to conjecture about likelihood of selection for this variant (Galvani and Slatkin 2003; Novembre et al. 2005; Stephens et al. 1998), other studies conclusively demonstrated that the deletion allele was present in prehistoric humans (Hummel et al. 2005), and has been subject to neutral evolution (Sabeti et al. 2005).

The protective effect of CCR5Δ32 has been confirmed in studies of children (Misrahi et al. 1998) and extended to demonstrate that children heterozygous for the variant also experience slower disease progression. Independent variants in the promotor region of *CCR5* (known as the *CCR5P1* haplotype) were observed to accelerate HIV disease progression (Martin et al. 1998), and this allele was shown to result in higher expression of CCR5 (McDermott et al. 1998). Genome-wide genotyping arrays do not universally contain variants that tag the CCR5Δ32 allele, but careful meta-analyses of published GWAS have readily replicated the effect of *CCR5Δ32* on reducing HIV acquisition and slowing disease progression, and the effect of *CCR5P1* on increasing HIV viremia (McLaren et al. 2013; McLaren et al. 2015). These meta-analyses also suggest that there may yet be additional signals in the region, not attributable to *CCR5Δ32* or *CCR5P1* that modify HIV control. These findings highlight the fact that the locus around CCR5 encodes several other chemokine receptors (Mummidi et al. 1998), including CCL3L1 (Dolan et al. 2007; Gonzalez et al. 2005) and CCR2 (Smith et al. 1997), which have been implicated in HIV control.

### Variation in HLA class I mediates altered HIV control through multiple mechanisms

Soon after the first description of HIV, cytotoxic T lymphocytes (CTL) targeting specific HIV antigens were identified in HIV-infected patients (Walker et al. 1987; Nixon et al. 1988). CTL were known, by this stage, to recognize target cells through virus-derived antigens presented by MHC class I molecules (Zinkernagel and Doherty 1974), known in humans as HLA. Contemporaneously, the exceptional polymorphism in HLA class I and class II was becoming more amenable to study at greater scale and at higher resolution using PCR methods to complement classical serological typing. The *HLA* loci on chromosome 6 are the most polymorphic genes of the human genome. *HLA-A*, *-B*, and *-C* (the class I genes) and *HLA-DR*, *-DP*, and *-DQ* (the class II genes) encode surface proteins that present host and pathogen-derived peptides to immune cells. The exceptional allelic diversity is mirrored by diversity in regulation of gene expression, and interaction with other components of the antigen presentation machinery and with other receptors. There is extensive linkage disequilibrium amongst *HLA* genes, particularly between *HLA-B* and *-C* genes in class I, and *-DR* and *-DQ* in class II.

The identification of CTL specific for HIV (Walker et al. 1987; Nixon et al. 1988) raised the possibility that the highly polymorphic *HLA* class I region may be involved in modifying HIV disease on the basis that such variation may alter CTL recognition. *HLA* alleles have been associated with more than 100 other diseases including most autoimmune and many infectious diseases, such as malaria and influenza (Matzaraki et al. 2017). Discoveries made in HIV have had broad implications for understanding immunity against viruses in general, as the *HLA* class I locus is the most robust correlate of HIV disease outcome in humans and has been widely replicated across settings and in GWAS. Here, we review several important themes that relate to the association between polymorphism in the *HLA* class I genes and HIV disease course (which has been the subject of other detailed reviews). Next, we highlight important, unanswered research questions.

#### The influence of HLA alleles on HIV disease progression includes both protective and harmful effects

**Fig. 2:**
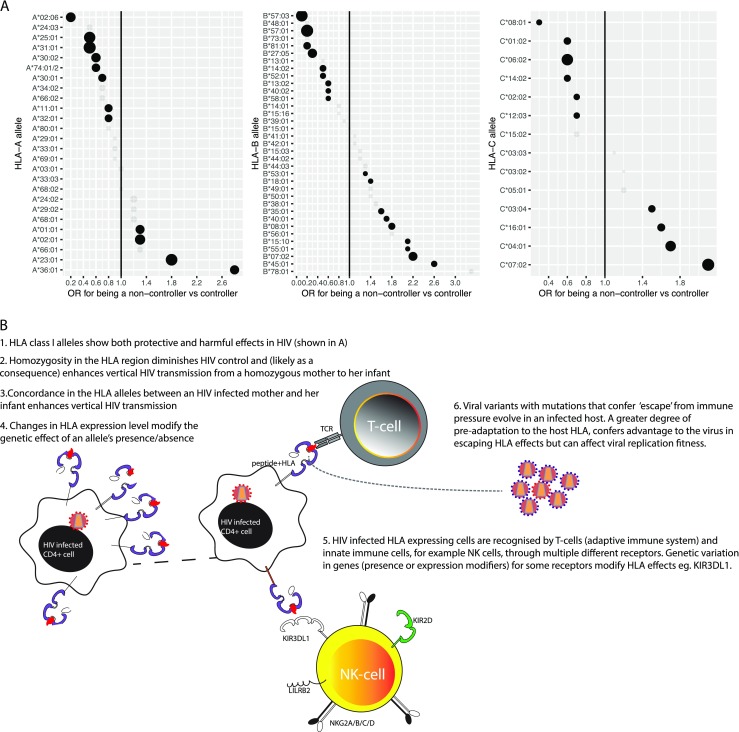
**a** The effect of *HLA* alleles on HIV vary across a continuum from harmful to protective effects and **b** involve a wide-variety of different mechanisms in mediating their effect. Data in **a** are derived from Bashirova et al. (2014)

Figure 2 shows the estimated effect of each *HLA-A*, *-B*, and *-C* allele from a stepwise logistic regression model comparing HIV controllers to HIV non-controllers (Bashirova et al. 2014). *HLA* class II alleles have vastly smaller effects than class I alleles, rarely reach significance after correction for multiple comparisons, and have been less well replicated. As shown, HLA-B*57, B*27, B*58:01, B*51, B*13, and B*81:01 are amongst the most reproducibly protective correlates of HIV. In contrast, HLA-B*58:02 and HLA-B*35Px alleles are associated with accelerated HIV disease progression. In addition, there is a wide range of alleles with intermediate effects. While some of the allelic effects on HIV appear reproducible across ethnic groups, there is likely considerable heterogeneity of effect by age, as is suggested by comparing effects in adults vs. children (Adland et al. 2015). For some alleles, temporal variation of the effect following seroconversion (Gao et al. 2005) has also been reported.

#### Reduced diversity in HLA is associated with poor HIV outcomes

Homozygosity at class I alleles has consistently been associated with poor HIV outcomes, including higher viremia, increased risk of transmission of HIV from an HLA-homozygote mother to her infant (likely due to higher viremia and higher likelihood of greater mother-child concordance), and accelerated HIV disease progression (Kroner et al. 1995; Carrington et al. 1999; Tang et al. 1999; Mackelprang et al. 2008). These data are consistent with a model in which the greater diversity of peptides presented to immune cells in heterozygotes is associated with a broader range of immune responses and better control, whereas homozygosity at the HLA loci constrains the set of peptides that can be presented. While homozygosity for HLA-Bw4 has been reported to be associated with enhanced HIV viremic control and reduced disease progression (Flores-Villanueva et al. 2001), this is almost certainly due to the fact that the most protective HLA class I alleles, which generally bind distinct HIV epitopes, carry the Bw4 epitope.

#### HLA concordance between HIV-discordant transmitting pairs is associated with elevated risk

Infants who share the same allele as their mother are at increased risk for acquiring HIV (Mackelprang et al. 2008; MacDonald et al. 1998; Polycarpou et al. 2002). This may have to do with the level of HIV adaptation to the *HLA* type carried by the infant.

#### Non-classical mechanisms play a role in HLA-allelic associations with HIV

The major mechanism through which *HLA* alleles have been shown to mediate their effects in HIV is the presentation of specific HIV-derived peptides to CTL, resulting in killing of HIV-infected target cells. This mechanism is incontrovertibly supported by functional studies that demonstrate HLA-restricted CTL targeting. However, variation in HLA expression level also appears to have a substantial effect. In particular, the effect of *HLA-C* alleles on HIV is inversely associated with the expression level of the allotypes they encode (Thomas et al. 2009; Apps et al. 2013). This finding was initially suggested by the identification of a genome-wide significant variant (rs924942) near *HLA-C* that was associated with HIV control (Fellay et al. 2007), a variant later shown to tag HLA-C expression in individuals of European descent. While rs924942 is highly unlikely to have any direct effect on HLA-C expression, in Caucasians, it marks variation in a microRNA-binding site in the 3′ UTR of HLA-C, which differentially affects HLA-C expression levels in an allele-dependent manner (Kulkarni et al. 2011; Kulkarni et al. 2013). It also marks an Oct1 binding site that has been shown to account for up to 35% of the differential expression level of HLA-C (Vince et al. 2016).

#### HLA class I alleles interact with other receptors; notably the killer cell immunoglobulin-like receptors (KIR) and leukocyte immunoglobulin-like receptors (LILR), further diversifying the function of HLA class I

The leukocyte receptor complex (LRC) on chromosome 19, and in particular the *KIR* locus, demonstrates pronounced structural and allelic variation. The *KIR* locus encodes anywhere from 9 to 16 surface receptors expressed on natural killer and T cells, which interact with HLA molecules to transduce either inhibitory or activating signals. Specific HLA allotypes interact with specific KIR; and in some cases, the peptide presented by the HLA allotype modifies this effect (*See* a previous review for further details (Martin and Carrington 2013)). The combination of *HLA-B* alleles that encode the Bw4 epitope with isoleucine at position 80 (Bw4-80I) and presence of *KIR3DS1* is associated with enhanced HIV control (Martin et al. 2002). Similarly, the combination of *HLA-B* and specific *KIR3DL1* alleles shows a gradient of effects on HIV, where *HLA-B Bw4* alleles with high expression *KIR3DL1* alleles show synergistic protective effects (Martin et al. 2007). Recent evidence has also demonstrated a role for LILR binding to HLA as a mediator of HLA effects against HIV. In this study, the strength of HLA-B binding to LILRB2, an inhibitory receptor expressed on myeloid and lymphoid cells that binds differentially to HLA-B in an allotype-dependent manner (Jones et al. 2011), is inversely associated with HIV viral control (Bashirova et al. 2014). HLA-B*35Px alleles in particular have been suggested to mediate their adverse impact on HIV through enhanced interaction and hence inhibition of LILRB2 expressing dendritic cells (Huang et al. 2009).

#### Focal viral sequence variation is a footprint of immune pressure

Evolution of viral variants that escape the targeted immune pressure mediated by HLA-restricted responses shows mutations specifically in epitopes presented by HLA. In a “genome-to-genome” analysis, Bartha et al. demonstrate that all of the variants in the HIV genome that are significantly associated with host genetic variants are also associated with variants in the HLA region (Bartha et al. 2013). Each of the major HIV proteins demonstrates HLA-associated selection pressure, although the pressure varies by protein (Carlson et al. 2012). These “escape variants” are enriched for localization within CTL epitopes, with 29% of all HLA-associated viral variants localizing within ±3 bp of an optimally described CTL epitope. Mutations that escape the CTL response occur most frequently at HLA anchor residues (1.8-fold enrichment) (Carlson et al. 2012). Just as *HLA* concordance between transmitting pairs has been observed epidemiologically to have adverse consequences, transmission of a virus even amongst *HLA*-discordant partners that is pre-adapted to the recipient *HLA* alleles is associated with impaired viremic control (Carlson et al. 2016). Not only does the *HLA* impact on HIV have an effect on the disease course in any given individual but also it shapes features of the epidemic at the population level. Increased immune pressure in individuals has been reported to reduce the virulence of the virus at a population level (Juarez-Molina et al. 2014; Payne et al. 2014; Herbeck et al. 2012; Kawashima et al. 2009). However, no clear or substantial decrease in HIV virulence has been observed since the epidemic began; and from the host standpoint, the detrimental effect of viral adaptation to the host HLA type appears greater than the beneficial effect associated with a loss in viral fitness (Carlson et al. 2016). Finally, although not as well replicated, carriage of specific KIR alleles is associated with variation at specific sites in HIV (Alter et al. 2011; Holzemer et al. 2015).

#### Outstanding research questions in HLA-HIV biology

1. What is the mechanism for the observed harmful effects of some HLA alleles? Protection conferred by certain *HLA* alleles is generally attributed to the HIV epitopes they recognize, though multiple characteristics of each allele are likely to explain their effects. *HLA* associations with susceptibility or impaired HIV control, on the other hand, are more difficult to explain although, once again, their overall effects are likely due to a combination of determinants. It is clear, however, that some alleles have detrimental effects and are not merely associated with *relative* impaired effects. For example, carriage of even one HLA-B*35 allele is associated with worse outcomes than being homozygous for any other *HLA-B* allele (Carrington et al. 1999). *HLA* associations with impaired clinical outcomes are common in autoimmune diseases, presumably due to involvement of that allele in instigating or maintaining autoimmune responses, but how this may be mediated in HIV is not immediately obvious. For some alleles, clear evidence of interaction with inhibitory LILRB2 receptors has been demonstrated, but this is unlikely to explain the full impact of an allele on poor outcome after HIV infection. Understanding this may yield new approaches for ameliorating the harmful effects of some *HLA* alleles.
2. Are *HLA* class II alleles reproducibly and independently associated with HIV control? A distinguishing feature of the nature of association in HIV compared to other diseases is the relative paucity of class II associations that predominate in autoimmune and other viral diseases. Although a handful of class II associations have been reported (Kroner et al. 1995; Ranasinghe et al. 2013; Julg et al. 2011), the extensive linkage disequilibrium in the class II region and relatively smaller sample sizes of class II-focused studies have made unequivocal demonstration of their role challenging. Even the largest GWAS have provided no indication that class II variation accounts for differential outcomes after HIV infection, though confounding circumstances may hamper identification of such effects (McLaren and Carrington 2015). Large studies in which *HLA* class I and linkage disequilibrium effects can be accounted for and where class II is typed directly may yield definitive results.
3. Are the effects of *HLA* alleles purely additive, or do some have synergistic effects, and how does this provide insights into mechanism? The observation that certain pairs of *HLA* class I alleles together may target sites that result in profound crippling of HIV (Dahirel et al. 2011; Ferguson et al. 2013) suggests that at least some combinations of alleles may have non-additive effects. The delineation of rules governing the effect of advantageous and deleterious features of *HLA* alleles when carried together requires large sample sizes, but if achievable, may inform the conditions under which non-additive effects on HIV control may be present and suggest epitopes that may be beneficial targets in vaccine design.
4. What additional non-classical mechanisms account for the association of HLA alleles with HIV control? Elevated HLA-C expression is robustly associated with enhanced HIV control, but the precise nature of this, including the relevance of KIR-mediated HLA-C recognition, remains unresolved. Understanding these effects may also help identify whether use of HLA-C/KIR blocking therapies, such as lirilumab may be useful in HIV. Although *HLA-B* alleles do not vary to any clear extent in mRNA expression (Ramsuran et al. 2017), recent studies demonstrate how *HLA-B* alleles vary in their impact on NK cell responses to HIV-infected cells (Merino et al. 2013). *HLA-B* alleles can be broadly split into two groups based on polymorphism encoding either a methionine (M) or threonine in the leader peptide of all *HLA-B* alleles. Horowitz et al. (2016) recently demonstrated that the ancestral M encoding variant almost invariably occurs on haplotypes with *HLA-Bw6* alleles and *HLA-C* alleles of the C1 group. They demonstrate that this results in profound differences in NK cell responses in vitro, and the effect of this variant in vivo may be an exciting avenue for further research. Finally, *HLA-A* expression levels vary substantially (Ramsuran et al. 2015) and whether such variation has any impact on HIV, as was shown for *HLA-C*, is an intriguing question.
5. Do *KIR-HLA* interactions involving *KIR2D* and *HLA-C* modify HIV outcomes in epidemiologic analyses (as do *KIR3DL1/S1* plus *HLA-Bw4*), and if so how? The interaction between HLA and KIR is incontrovertible, yet the in vivo effects of this complex interaction have been challenging to study because of the marked polymorphism in the two regions. Viral sequence footprints indicative of KIR-mediated pressure and in vitro evidence of an effect on HIV responses are suggestive of further epidemiological associations and in-depth examination of these two regions together in large datasets where there is power to control for confounding by known *HLA*/*KIR* effects may prove fruitful. Moreover, the originally reported *HLA-Bw4-80I*/*KIR3DS1* associations deserve re-appraisal in light of the evidence that KIR3DS1 binds HLA-F (Burian et al. 2016; Garcia-Beltran et al. 2016), and current lack of clarity on whether KIR3DS1 may also bind to HLA-Bw4 epitopes in vivo under specific circumstances, such as in the presence of specific peptides (O'Connor et al. 2015).

##### Immunogenetics of HIV on the horizon

A crucial caveat to the extant knowledge on genetic modifiers of HIV is that genetically complex regions are systematically under-represented. For example, the evidence for *KIR-HLA* interactions undoubtedly suggests that additional effects are likely to exist, but are not yet identified because they are neither tagged by single-nucleotide variants in GWAS studies nor have they been fully characterized. For example, aside from *KIR3DL1*, allelic typing of *KIR* loci has not been widely performed, but rather, the field has concentrated primarily on associations at the gene presence/absence level. New, in-depth typing methods for the KIR locus will facilitate progress in this regard (Norman et al. 2016). Similarly, variation in the *immunoglobulin* locus and *TCR* loci, which are fiendishly complex, may be fruitful, as these loci may have substantial effects in HIV given the known importance of binding and neutralizing antibody responses in HIV.

The relative absence of genetic correlates of disease outside of *HLA* and *CCR5* juxtaposes against the extensive evidence for a slew of host proteins involved in HIV pathogenesis. This may be due in some cases to their lack of functional polymorphism or to the exquisite redundancy that is characteristic of many of these biological systems. Also, pairs of variants that affect functionally linked components may be expected to show epistatic associations, and these would not be identified by classical GWAS techniques. Examples where *HLA* in combination with *KIR3DS1*, *KIR3DL1*, and *LILRB2* are highly suggestive that such genetic effects do occur at some frequency. We anticipate that careful analysis of such pairs (or higher order combinations), guided by the extensive biological characterization of host restriction or dependency factors identified by siRNA (Bushman et al. 2009; Zhu et al. 2014) and in newly published CRISPR screens (Park et al. 2017), will be fruitful.

A third exciting prospect is the use of Mendelian randomization approaches to validate or refute non-genetic correlates of disease. For example, immune activation has long been linked to HIV acquisition, is associated with HIV disease progression, and has some mechanistic basis for being causally involved in these associations (Sereti and Altfeld 2016). However, clinical trial efforts to modify immune activation have not had a profound effect on HIV (Bandera et al. 2017). To distinguish whether such efforts have been unsuccessful due to an insufficient ability to reduce immune activation, or whether immune activation is an epiphenomenon of HIV disease, Mendelian randomization studies may be fruitful. Using genetic correlates of immune activation as an instrument could allow inference as to the causal role of immune activation in HIV disease. This is done by specifically identifying genetic variants associated with immune activation, and then testing whether these variants are associated with HIV outcomes to the extent predicted by the effect of the variant on immune activation and the reported association between activation and outcomes (Didelez and Sheehan 2007). The major advantage of such an approach is its use of genetic variables, which are randomly inherited and subject to fewer confounders. Such efforts have been immensely powerful, for example, in refuting HDL as a target for cardiovascular preventive therapy. Studies have demonstrated that HDL cholesterol levels are unlikely to be causally linked to cardiovascular outcomes (Voight et al. 2012) because variants that robustly associate with HDL cholesterol are not associated with outcomes.

## Concluding remarks

Our present understanding of genetically mediated control of HIV acquisition and disease progression stands amongst the most advanced of any polygenic disorder, and this holds true in comparison to malaria or tuberculosis, the two other leading infectious pandemics of our time. The two loci originally identified by candidate gene studies in the 1990s (*CCR5* and *HLA*) remain the only replicable and robust correlates of HIV acquisition or of disease progression. The observation that homozygotes for the *CCR5∆32* null mutation are highly resistant to HIV infection and that heterozygotes for this mutation progress more slowly to disease has led to targeted therapy in the form of approved CCR5 antagonists such as maraviroc, contributed to the only HIV cure in an adult recipient of a CCR5Δ32 allogeneic stem cell transplant (Hutter et al. 2009), and has been leading targets for genome-editing strategies in HIV (Tebas et al. 2014). Although direct translation of *HLA* findings into patient care is less straightforward, the understanding of the central role of HLA-restricted responses and the findings that stemmed from this, such as the importance of gag-specific responses in vivo, is playing an important role in antigen prioritization in HIV vaccine development. Nevertheless, the nature of how *HLA* variation affects HIV continues to be expanded upon. Modern genetics provides a powerful tool to determine causal pathways involved in HIV control, and discoveries regarding the many layers of *HLA* effects on HIV disease have and will continue to have major relevance to our understanding of human disease in general.

## Electronic supplementary material

Supplementary Table 1 Summary of findings of GWAS studies of HIV outcomes or HIV-related outcomes extracted from the GWAS catalog (https://www.ebi.ac.uk/gwas/). (XLSX 74 kb).
